# The role of *Candida albicans* in root caries biofilms: an RNA-seq analysis

**DOI:** 10.1590/1678-7757-2019-0578

**Published:** 2020-04-27

**Authors:** Laís Daniela EV, Nailê DAMÉ-TEIXEIRA, Thuy DO, Marisa MALTZ, Clarissa Cavalcanti Fatturi PAROLO

**Affiliations:** 1 Universidade de Federal do Rio Grande do Sul Faculdade de Odontologia Departamento de Odontologia Preventiva e Social Porto AlegreRio Grande do Sul Brasil Universidade de Federal do Rio Grande do Sul , Faculdade de Odontologia , Departamento de Odontologia Preventiva e Social , Porto Alegre , Rio Grande do Sul , Brasil .; 2 Universidade de Brasília Faculdade de Ciências da Saúde Departamento de Odontologia BrasíliaDistrito Federal Brasil Universidade de Brasília , Faculdade de Ciências da Saúde , Departamento de Odontologia , Brasília , Distrito Federal , Brasil .; 3 University of Leeds Faculty of Medicine & Health School of Dentistry Leeds United Kingdom University of Leeds , Faculty of Medicine & Health , School of Dentistry , Leeds , United Kingdom .

**Keywords:** Sequence analysis, RNA, Candida albicans, Root caries, Transcriptome

## Abstract

**Objective:**

This study sought to analyze the gene expression of *Candida albicans* in sound root surface and root caries lesions, exploring its role in root caries pathogenesis.

**Methodology:**

The differential gene expression of *C. albicans* and the specific genes related to cariogenic traits were studied in association with samples of biofilm collected from exposed sound root surface (SRS, n=10) and from biofilm and carious dentin of active root carious lesions (RC, n=9). The total microbial RNA was extracted, and the cDNA libraries were prepared and sequenced on the Illumina Hi-Seq2500. Unique reads were mapped to 163 oral microbial reference genomes including two chromosomes of *C. albicans* SC5314 (14,217 genes). The putative presence of *C. albicans* was estimated (sum of reads/total number of genes≥1) in each sample. Count data were normalized (using the DESeq method package) to analyze differential gene expression (using the DESeq2R package) applying the Benjamini-Hochberg correction (FDR<0.05).

**Results:**

Two genes (CaO19.610, FDR=0.009; CaO19.2506, FDR=0.018) were up-regulated on SRS, and their functions are related to biofilm formation. Seven genes ( *UTP20* , FDR=0.018; *ITR1* , FDR=0.036; *DHN6* , FDR=0.046; *CaO19.7197* , FDR=0.046; *CaO19.7838* , FDR=0.046; *STT4* , FDR=0.046; *GUT1* , FDR=0.046) were up-regulated on RC and their functions are related to metabolic activity, sugar transport, stress tolerance, invasion and pH regulation. The use of alternative carbon sources, including lactate, and the ability to form hypha may be a unique trait of *C. albicans* influencing biofilm virulence.

**Conclusions:**

*C. albicans* is metabolically active in SRS and RC biofilm, with different roles in health and disease.

## Introduction

The bacterial biofilm associated with root caries lesions must harbor microorganisms that can produce acid from carbohydrates (acidogenicity) and must be able to growth in a low-pH environment (aciduricity). ^1^ Diverse bacteria are prevalent and involved in the etiology of root caries, albeit to date, little has been explored regarding other microorganisms domains, such as archea, fungi and virus, and their role in biofilms. Previous studies demonstrated that *Streptococcus mutans* , *Lactobacillus* species (spp.), and *Veillonella* spp., as well as *C. albicans,* are present in major proportion in root caries than in sound root surface. ^2^ *Actinomyces* spp., *Veillonella* spp., *Streptococcus* spp., *Bifidobacterium* spp., *Rothia, Enterococcus* , *Staphylococcus* spp., *Capnocytophaga* spp., *Prevotella* spp. and *Candida* spp., were also cultivated from root caries. ^1 , 3 , 4^

*Candida* species has been associated with dental caries, especially with early childhood caries and root caries. ^5^ A strong association was found between the prevalence of *C. albicans* and dental caries. ^6^ Several authors showed that the proportion of *Candida* species was higher in individuals with caries than in individuals without caries. Furthermore, *C. albicans* is an important colonizer of carious lesions and has been found frequently in dentin caries lesions rather than in biofilm or saliva. ^4^ Lower salivary flow rate, a common occurrence in older adults, is one of the factors that promote favorable conditions for a presence of *C. albicans* in these sites. ^7^ However, it is still unknown whether the yeast acts as caries pathogen or plays a role as a commensal microbe. *C. albicans* possess some important properties that can characterize it as an important root caries pathogen. It is capable of adhering to saliva-coated hydroxyapatite and possesses strong adherence to collagen. ^8^ It is as acid tolerant and acidogenic as *S. mutans* and *Lactobacilli* , which are both well-established cariogenic pathogens. ^9^ To determine the role of *C. albicans* in root caries, a high-throughput sequencing of mRNA (RNA-Seq) was applied in clinical biofilms samples from two distinct conditions: sound root-surface biofilms and root carious lesions biofilms. This technique may be helpful to investigate *Candida’s* role in a carious biofilm.

## Methodology

This study is part of the project “metatranscriptome of root caries”. ^10^ Briefly, volunteers to this study were divided into two groups: sound exposed root surface group (SRS; n=10) and root caries group (RC; n=30). Participants were allocated to the SRS group (n=10) if they had an exposed root surface on at least one tooth and no root caries lesions. Dental biofilms were collected with sterilized Gracey curette from all available exposed root surfaces. The number of exposed root surfaces varied among individuals. Participants recruited to the root caries (RC) group ( *n* =30) had one primary cavitated root lesion in need of restorative treatment. All lesions showed characteristics of present activity (soft and yellow dentin). Biofilm and carious dentin samples (of soft and infected tissue) were collected from patients during the restorative treatment. All participants were asked to refrain from tooth brushing for at least 12 hours prior to the sampling, to allow for dental biofilm accumulation, and were also asked to refrain from eating and drinking for at least 1 hour prior to the sampling. After collection, biofilm and carious dentin were immediately placed in 1 mL of RNA protect reagent (Qiagen, Hilden, North Rhine-Westphalia, Germany). The total RNA was extracted using the UltraClean ^®^ Microbial RNA Isolation (Mo-bio, San Diego, Califórnia, USA) with on-column DNase digestion (Qiagen, Hilden, North Rhine-Westphalia, Germany). Samples with total RNA concentration <30 ng/RNA were pooled, leading to a final sample size of 10 SRS and 9 RC. The Ribo-Zero™ Meta-Bacteria Kit (Illumina, Madison, Wisconsin, USA) was used for mRNA enrichment and Illumina ^®^ TruSeq™ library prep protocols (Illumina, San Diego, Califórnia, USA) were used to library preparation and sequencing was performed with Illumina HiSeq2500 (Illumina, San Diego, Califórnia, USA). RNA sequencing data are available in the National Center for Biotechnology Information (NCBI) Sequence Read Archive, under the accession numbers SRS779973 and SRS796739. FASTQ files were obtained for each sample and imported into the CLC Genomics Workbench 7.5.1 software (CLC bio, Aarhus, Denmark) for mapping against 163 oral microbial genomes. ^10^ The number of sequence reads that have been assigned to each gene is considered as the read count data.

### *Candida albicans* genome and data analysis

The *C. albicans* SC5314 was the genome selected for this study. This strain was chosen for being largely studied and its genome has been fully sequenced as well. After mapping, a count table was generated containing the read count for 14,217 oral *C. albicans* SC5314 genes.

The putative presence of the organism in the sample was estimated by the sum of reads assigned to *C. albi* cans divided by the total number of genes for each sample. Samples with ≥14,217 reads were considered as valid; then samples with less than 30% of genes with at least one read were excluded from the analysis.

The number of reads and the relative median expression (RME) (25 ^th^ -75 ^th^ ) level for genes were estimated for each of the sample groups, as previously described. ^11^ Then, the RME was ranked to observe the most highly expressed transcripts in RC and SRS samples. To draw a profile of gene expression, the median of RME of transcripts in SRS and RC conditions were considered low expression RME between 0-10, medium 11-100, and high above 100 (percentile 10 of RME distribution). RME was calculated from the median values of normalized read counts using DE-Seq algorithm. Genes related to *C. albicans* virulence factors were analyzed: invasion, biofilm formation and co-aggregation, adherence and damage, morphogenesis, acid production, acid tolerance and stress response.

All RME medians for SRS and RC were ranked and all genes with median RME values ≥100 *per* group were analyzed for an overview of the most prevalent genes.

Differential gene expression was inferred between sample groups by applying the R package DESeq2. ^12^ The cut-off for designating a gene as being differentially expressed was a change in transcript levels of at least 2-fold change (Log2FoldChange>1) and false discovery rate (FDR) <0.05 (padj value<0.05, Benjamini & Hochberg). Functions and putative pathogenicity in root caries of genes up-regulated in SRS and RC were analyzed.

Regarding to ethics considerations, this study was approved by the Federal University of Rio Grande do Sul research ethics committee (process n° 427.168) and by the research ethics committee of the National Research Ethics Service Committee Yorkshire & The Humber – Leeds West (protocol no. 2012002DD). All volunteers signed an informed consent form and received clinical dental assistance.

## Results

**Figure 1:**
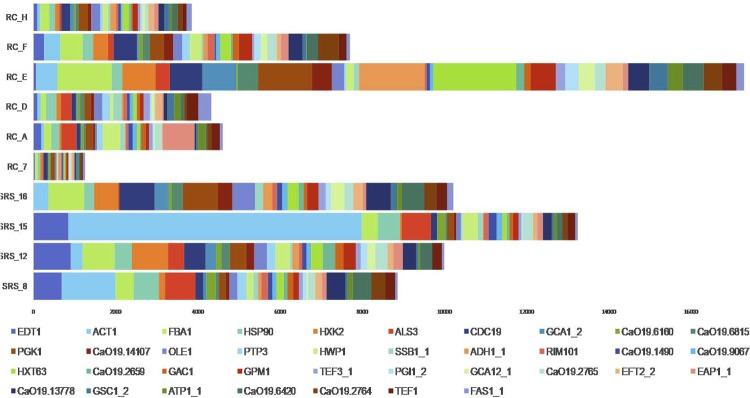
Relative median expression (RME; log10) of genes in the Sound Root Surfaces (SRS; n=4) and Root Caries (RC; n=6) samples. RME was calculated from the median values of normalized read counts. The top median RME values for SRS and RC were selected and sorted, and indicate the most expressed genes by *C. albicans* SC5413

According to the cut-off point chosen to determine the putative presence of a mapped organism in each sample, *C. albicans* was present in n=4 biofilms from SRS and in n=6 biofilm from RC, as shown in Figure 1 . Table 1 shows that the number of reads distribution in sound and disease samples were equal (p=0.522).

**Table 1:** Total numbers of DESeq normalized reads (median/percentile/range) by group

	Median	25 ^th^ -75 ^th^	Range
SRS	157.175	48.329 - 197.776	22.721 – 223.511
RC	209.495	79.770 – 571.876	48.759 – 738.400

*SRS =sound root surface. RC=root caries

### Gene expression *per* sample

Figure 1 shows an overview of the most prevalent genes in *C. albicans* biofilm with and without caries. A total of 37 genes with median of RME>100 were analyzed (34 in SRS and 20 in RC). A total of 17 genes have RME>100 in both health and disease conditions for all the samples ( *FBA1, HSP90, HXK2, ALS3, CDC19, PGK1, OLE1, HWP1, HXT63, GPM1, CaO19.2765, EFT2_2, EAP1_1, CaO19.13778, CaO19.6420, CaO19.2764* and *TEF1* ), wheras 17 genes were expressed only in SRS ( *EDT1, ACT1, GCA1_2, CaO19.6160, CaO19.6815, CaO19.14107, PTP3, SSB1_1, ADH1_1, RIM101, CaO19.1490, CaO19.9067, CaO19.2659, GAC1, TEF3_1, PGI1_2* and *GCA12_1* ) and just 3 genes were expressed only in RC conditions ( *GSC1_2, ATP1_1* and *FAS1_1* ), as shown in Table 2 .

**Table 2:** Relative median expression (RME) and percentiles (25 th -75 th ) of genes related to virulence factors in *Candida albicans* in the Sound Root Surfaces (SRS; n=4) and Root Caries (RC; n=6) samples

Accession ID	Median SRS (25 ^th^ -75 ^th^ )	Median RC (25 ^th^ -75 ^th^ )	Virulence Trait
**ACT**			
ACT1_1	416.13(156.10-2871.60)	38.92(2.07-221.80)	Invasion
ACT1_2	415.74(147.50-2811.20)	37.65(2.06-209.30)	
ACT2_1	3.75(0.93-3.85)	8.21(2.82-12.75)	
ACT2_2	5.02(4.02-9.45)	6.81(6.69-10.22)	
**LIP9**			
LIP9_2	0(0-0)	0.47(6.36-1.85)	Invasion
LIP9_1	0(0-0)	0(6.10-1.22)	
**PLB2**			
PLB2_1	0(0-0.47)	0.95(0-7.49)	Invasion
PLB2_2	0(0-0)	0.18(0-4.16)	
**TEC1**			
TEC1_1	21.98(18.5-26.30)	14.46(2.65-26.40)	Biofilm Formation
TEC1_2	18.23(6.75-21.38)	12.08(6.96-37.16)	
**EFG1**	70.10(38.95-86.02)	22.14(0-62.93)	Biofilm Formation/ Morphogenesis
**HWP1**			
HWP1_1	127.29(19.59-176.94)	80.53(0-167.24)	Biofilm Formation/ Adherence
HWP1_2	126.31(20.02-188.10)	72.18(0-148.94)	
**ALS1**			
ALS1_1	9.26(0.78-36.07)	26.58(0-84.96)	Adherence
ALS1_2	39.03(13.44-64.22)	22.85(0-147.17)	
**ALS2**			
ALS2_1	4.61(0.93-13.47)	4.64(0.78-6.23)	Adherence
ALS2_2	2.79(0.62-9.47)	3.73(0-7.46)	
**ALS3**			
ALS3_1	199.32(45.47-274.46)	88.26(0-122.72)	Invasion/Adherence
ALS3_2	50.15(3.16-98.69)	33.79(0-77.51)	
ALS3_3	171.47(34.94-255.39)	91.19(2.51-124.35)	
ALS3_4	159.55(34.17-225.69)	24.39(1.47-89.82)	
**ALS4**	5.93(1.04-13.48)	10.63(0-23.22)	Adherence
**ALS5**			
ALS5_1	3.47(2.07-4.99)	5.04(0-11.62)	Adherence
ALS5_2	1.35(0.16-3.42)	5.65(0-12.03)	
**ALS6**	2.76(0-6.47)	9.35(0-17.69)	Adherence
**ALS7**	9.38(6.41-37.42)	18.73(0-36.87)	Adherence
**ALS9**			
ALS9_1	3.80(0-11.14)	4.03(0-13.27)	Adherence
ALS9_2	6.48(1.35-9.75)	2.30(0-8.02)	
ALS9_3	0(0-0)	1.80(0-4.62)	
**RBT5**			
RBT5_1	51.14(18.72-76.96)	38.22(0-70.20)	Adherence
RBT5_2	43.62(15.26-72.99)	33.44(0-74.34)	
**SAP1**			
SAP1_1	0(0-0.27)	1.30(0-2.84)	Collagen degradation
SAP1_2	0(0-0.52)	0(4.06-2.01)	
**SAP2**			
SAP2_1	0.31(0-1.19)	0.93(55.86-4.55)	Collagen degradation
SAP2_2	0.35(0-4.2)	1.18(115.19-4.03)	
**SAP3**			
SAP3_1	0(0-0)	0(26.17-1.93)	Collagen degradation
SAP3_2	0(0-0.27)	0.36(29.39-2.27)	
**SAP4**			
SAP4_1	0(0-0)	0(0-0.61)	Collagen degradation
SAP4_2	0(0-0)	1.65(0-4.38)	
**SAP5**			
SAP5_1	0(0-0.27)	0.41(1.16-3.76)	Collagen degradation/ Biofilm formation/Invasion
SAP5_2	0(0-0)	1.85(0-3.56)	
**SAP6**			
SAP6_1	0(0-0)	0.59(0.52-3.61)	Collagen degradation
SAP6_2	0(0-0)	1(0-6.06)	
**SAP7**			
SAP7_1	16.52(14.19-21.6)	11.59(6.80-28.20)	Collagen degradation
SAP7_2	18.89(6.9-25.58)	15.71(0-25.99)	
**SAP8**			
SAP8_1	0(0-4.03)	1.295(0-4.85)	Collagen degradation
SAP8_2	0(0-0)	0.06(0-3.67)	
**SAP9**			
SAP9_1	13.75(5.16-18.51)	16.32(0.54-25.66)	Collagen degradation
SAP9_2	16.27(5.08-25.63)	17.15(1.07-30.05)	
**SAP10**			
SAP10_1	0.31(0-0.94)	1.84(4.84-6.65)	Collagen degradation
SAP10_2	0(0-0.27)	1.06(13.5-3.99)	
**SAP98**			
SAP98_1	0.31(0-0.94)	0.52(0.35-2.27)	Collagen degradation
SAP98_2	0(0-0)	0.53(1.92-1.64)	
**SAP99**			
SAP99_1	0(0-0)	1.24(0-4.56)	Collagen degradation
SAP99_2	0.18(0-1.01)	0.12(0-1.93)	
**CDC24**			
CDC24_1	6.25(1.13-13.59)	9.45(1.21-13.71)	Morphogenesis
CDC24_2	6.66(4.24-11.23)	5.8(0-12.67)	
**CDC42**			
CDC42_1	6.58(1.99-11.37)	3.45(0-4.37)	Morphogenesis
CDC42_2	1.38(0-3)	2.95(0-3.9)	
**STE11**			
STE11_1	9.07(5.47-17.16)	7.33(0.54-14.34)	Morphogenesis
STE11_2	14.44(4.44-28.53)	7.30(2.34-18.04)	
**CST20**			
CST20_1	16.77(8.48-24.6)	12.58(0-23.91)	Morphogenesis
CST20_2	16.99(12.72-21.04)	15.66(0-22.24)	
**HST7**			
HST7_1	6.02(5.50-7.33)	4.55(0-11.04)	Morphogenesis
HST7_2	5.06(1.24-5.33)	3.73(0-9.39)	
**CYR1**			
CYR1_1	5.06(1.24-9.98)	4.47(0-13.31)	Morphogenesis
CYR1_2	8.35(5.96-10.10)	7.98(0-17.23)	
**TPK2**			
TPK2_1	6.71(5.57-7.61)	5.79(0-9.88)	Morphogenesis
TPK2_2	5.64(1.13-7.49)	7.98(0-10.83)	
**PKA1**			
PKA1_1	1.85(0-3.82)	2.04(24.88-3.86)	Morphogenesis
PKA1_2	15.85(8.48-21.81)	6.53(15.73-9.34)	
**CZF1**			
CZF1_1	0.52(0-2.11)	1.27(5.05-5.85)	Morphogenesis
CZF1_2	1.75(0.26-4.65)	1.35(6.26-3.44)	
**NRG1**	2.28(0.64-4.99)	6.05(6.01-13.69)	Morphogenesis
**CPH1**			
CPH1_1	9.58(1.56-35.08)	8.13(0-17.53)	Morphogenesis
CPH1_2	0(0-0.47)	0.59(0-1.48)	
**CDC28**			
CDC28_1	0.35(0-1.56)	1.69(0-3.16)	Morphogenesis
CDC28_2	1.04(0-8.58)	2.83(0-6.96)	
**CPH2**			
CPH2_1	52.42(21.47-56.66)	23.81(0-46.74)	Morphogenesis
CPH2_2	26.53(14.35-43.96)	13.48(0-20.62)	
**HSP90**			
HSP90_1	212.35(129.29-284.69)	89.73(19.53-129.49)	Morphogenesis/ Stress Response
HSP90_2	254.89(158.85-313.28)	111.99(14.61-143.75)	
**RAS1**			
RAS1_1	2.59(0-5.46)	5.45(0-6.90)	Morphogenesis
RAS1_2	3.30(0.62-5.07)	4.68(0-5.76)	
**TUP1**			
TUP1_1	24.91(19.85-32.21)	10.47(0-14.82)	Morphogenesis
TUP1_2	20.69(9.82-28.32)	8.03(0-10.93)	
**RFG1**			
RFG1_1	0(0-1.04)	2.28(0-6.62)	Morphogenesis
RFG1_2	7(1.35-8.9)	5.59(0-11.98)	
**HYR1**			
HYR1_1	0(0-0.47)	0.47(0-1.67)	Morphogenesis
HYR1_2	0(0-0.47)	1.42(0-7.26)	
**ECE1**			
ECE1_1	0.18(0-2.40)	0.42(0-1.93)	Morphogenesis
ECE1_2	1.23(0-4.65)	1.03(0-4.44)	
**PHR1**			
PHR1_1	33.62(25.20-94.04)	38.18(0-57.07)	Acid tolerance
PHR1_2	36.96(27.23-104.02)	48.26(0-57.07)	
**PHR2**			
PHR2_1	4.71(2.27-29.58)	7.77(0.78-32.06)	Acid tolerance
PHR2_2	51.11(2.69-118.06)	58.10 (0.18-137.34)	
**RIM101**	120.39(89.45-151.32)	65.67(2.72-111.54)	Stress response/ Morphogenesis
**HOG1**			
HOG1_1	4(1.89-5.07)	4.36(1.30-5.09)	Stress response
HOG1_2	3.96(0.77-6.99)	4.2(1.065-7.63)	
**CAP1**			
CAP1_1	7.15(1.73-7.64)	5.21(3.12-8.725)	Stress response
CAP1_2	11.01(6.34-14.78)	9.25(1.65-11.7)	

### Expression of genes related to possible cariogenic traits

*C. albicans* genes associated with possible virulence factors (RME and percentiles of these genes) were evaluated in both conditions ( Table 2 ). We found transcript of 51 out of 67 genes related to virulence traits that are presented in the literature as important factors. None of these genes had significant differential expression.

### Differential expression analysis (DE)

**Figure 2:**
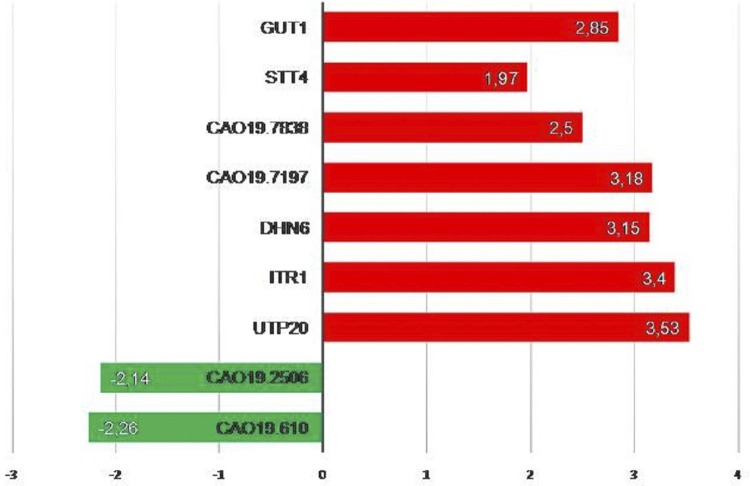
Differential expression (Log2FoldChange) of genes up-regulated in sound root surface (SRS; negative values, green bars) and up-regulated in root caries (RC, positive values, red bars) calculated using DESeq2 algorithms. FDR<0.05. GUT1= potential glycerol kinase; STT4= hypothetical protein phosphatidylinositol-4-kinase; CaO19.7838= flocullin-like protein serine-rich; CaO19.7197= hypothetical protein; DHN6= dehydrin hypothetical protein; ITR1= potential Myo-inositol transporter; UTP20= potential U3 small nucleolar RNAs protein; CaO19.2506= hypothetical protein with a very weak similarity to Streptoccal proline-rich surface protein PspC; CaO19.610= potential DNA binding regulator of filamentous growth

The DE analysis has shown the overexpressed genes in root biofilms with and without caries ( Figure 2 ). The up-regulated genes in SRS group were *CaO19.610* and *CaO19.2506* . The *CaO19.610* (FDR=0.009) codes for a potential DNA binding regulator of filamentous growth. The *CaO19.2506* (FDR=0.018) codes for a hypothetical protein with a very weak similarity to *Streptococcal* proline-rich surface protein PspC.

The up-regulated genes in RC group were *UTP20, ITR1, DHN6, CaO19.7197, CaO19.7838, STT4,* and *GUT1* . The *UTP20* (FDR=0.018) codes for a potential U3 small nucleolar RNAs (snoRNA) protein. The *ITR1* (FDR=0.036) codes for a potential active sugar transporter, potential Myo-inositol transporter, similar to *S. cerevisiae* ITR1 (YDR497C). The *DHN6* (FDR=0.046) codes for a dehydrin hypothetical protein. The *CaO19.7197* (FDR=0.046) codes for a hypothetical protein similar to *S. cerevisiae* YLR002C, with unknown function. The *CaO19.7838* (FDR=0.046) codes for a flocculin-like protein serine-rich, repetitive ORF similar to *S. cerevisiae MUC1* (YIR019C) cell surface flocculin. The *STT4* (FDR=0.046) codes for a hypothetical protein phosphatidylinositol-4-kinase. The *GUT1* (FDR=0.046) codes for a potential glycerol kinase Gut1p, likely carbohydrate kinase similar to *S. cerevisiae* GUT1 (YHL032C) glycerol kinase.

## Discussion

Possible virulence traits of *Candida* spp. were related to several survival strategies such as the capacity to exploit and invade the host tissues, forming biofilms and co-aggregate to various microorganisms, switching form, producing acids and reacting to stress. *C. albicans* is metabolically active in biofilm of SRS and RC, presenting different roles in health and disease. Some genes were expressed in both conditions, which seem to be relevant to *C. albicans* survival to these sites. Genes overexpressed in SRS were involved in biofilm formation, while genes overexpressed in RC were involved in survival strategies that could be related to cariogenicity.

Two genes were up-regulated in SRS biofilms. The *CaO19.610* codes for a potential DNA binding regulator of filamentous growth. This gene is a version of *C. albicans efg1* with altered C terminus. EFG1 protein is a key transcriptional regulator in *C. albicans* and controls various aspects of morphogenesis and metabolism ^13^ , being required for the true hyphae growth, biofilm formation, cell adhesion and filamentous growth in *C. albicans* . ^14^ *Efg1* gene confers to *C. albicans* the capacity of transition from commensal microorganism to opportunistic pathogen status. ^15^ In an *in vitro* experiment, *efg1* had significantly higher gene expression at initial biofilm formation stage. ^16^ Other studies showed that EFG1 is essential for the formation of a mature and stable bioﬁlm that is resistant to antifungal therapy and to immune system, allowing the colonization of the root site. ^17 , 18^ The *CaO19.2506* codes for a hypothetical protein with a very weak similarity to streptococcal proline-rich surface protein PspC. In *S. pneumoniae* , PspC has a well-established importance in adherence and colonization. ^19^ The possible function of *CaO19.2506* is related to adhesion and coding for a membrane adhesin. Both filamentous growth and cell wall adhesion are important in biofilm formation and are required for the establishment of *C. albicans* in root surfaces biofilm. These characteristics could explain why *C. albicans* has been largely observed colonizing sound root surface. ^5^

The role of *C. albicans* in root caries could be potentially more complex. Seven genes were up-regulated in root caries conditions expressing different functions. The *CaO19.7197* codes for a hypothetical protein similar to *S. cerevisiae* YLR002C, with unknown function. Several hypothetical proteins and genes with uncharacterized function were identified in this study, highlighting the importance of more studies related to *C. albicans* transcriptome. The *DHN6* codes for a dehydrin hypothetical protein, related to stress tolerance in plants. These proteins can be induced in vegetative tissues by different stress factors that cause cell dehydration (i.e., drought, salinity, cold, heat, low temperature, etc). ^20 , 21^ The *STT4* codes for a hypothetical protein phosphatidylinositol-4-kinase. The gene *STT4* is essential for viability and plays an important role in the phosphatidylinositol-mediated signal transduction pathway required for cell wall integrity. ^22^ Therefore, the up-regulated genes *DHN6* and *STT4* could be related to the ability to survive in an extreme environment with several stress factors (low pH, carbohydrate viability, for example) such as the ones found in root cavitated caries lesions. The *UTP20* codes for a potential U3 small nucleolar RNAs (snoRNA) protein. *UTP20* has been reported as a component of U3 snoRNA protein complex and has been implicated in 18S rRNA processing, being essential for 18 rRNA function. ^23 , 24^ The *ITR1* codes for a potential active sugar transporter, potential Myo-inositol transporter, similar to *S. cerevisiae ITR1* (YDR497C). Myo-inositol is an essential substrate for *C. albicans* , and it can be used as carbon source. For its survival, *C. albicans* must be able to synthesize the essential metabolite inositol or acquire it from the host. *C. albican* s could not transport inositol and become nonviable in the absence of *ITR1* . ^25^ The CaO19.7838 codes for flocculin-like protein serine-rich, repetitive ORF similar to *S. cerevisiae MUC1* (YIR019C) cell surface flocculin. *MUC1* encodes cell-surface flocculin and it is required for pseudohyphal and invasive growth of *C. albicans.* ^14^ The up-regulation of this invasive growth gene shows the importance of this virulence trait for the colonization/penetration of *C. albicans* in the carious dentin. The *GUT1* codes for a potential glycerol kinase Gut1p, likely carbohydrate kinase similar to *S. cerevisiae GUT1* (YHL032C) glycerol kinase (NCBI). In *Saccharomyces cerevisiae* , glycerol utilization is mediated by two enzymes, glycerol kinase (Gut1p) and mitochondrial glycerol-3-phosphate dehydrogenase (Gut2p). The carbon source regulation of *GUT1* depends on carbon source availability. The promoter activity of *GUT1* was lower during growth on glucose and highest on the non-fermentable carbon sources, glycerol, ethanol, lactate, acetate and oleic acid. ^26^ *UTP20, ITR1, CaO19.7838* , and *GUT1* are genes related to *C. albicans* metabolism associated with caries progression to a cavitated status.

The overexpressed genes in RC were related to sugar transport *(ITR1* – Myo-inositol) and to carbon source regulation ( *GUT1* – Glycerol kinase), that were related to the use of alternative carbon sources ( Figure 2 ). The use of lactate by *C. albicans* could be related to the pH regulation (neutralization) in biofilm, which is important for the microbiome survival. ^27^ Furthermore, this neutralization of the medium could explain the *CaO19.7838* overexpression, a gene related to hyphal growth since hyphal formation is stimulated by neutral pH. ^28^ Morphogenesis is a special virulence trait of *C. albicans,* and hyphal form is related to pathogenesis, being more invasive and contributing to host tissue damage ^29 , 30^ , as well as contributing to the active cavitation of RC lesions. Besides the stimulation of hyphal growth, changes in carbon source has a significant impact on the *C. albicans* virulence, resulting in an increased resistance to stresses, adherence, biofilm formation, drug resistance, and immune recognition when compared with glucose-grown cells. ^31^ Although a cariogenic environment is related to low pH conditions, the excessive production of acids could affect the biofilm metabolism. For the cariogenic biofilm survival, it is important to have a microorganism that main the viability of the biofilm, thus preventing excessive acidification even in a carious habitat.

## Conclusions

Our data shows that *Candida albicans* SC5314 have an active metabolism in biofilm of SRS and biofilm of carious dentin of RC as well. The differential expression analysis shows that, in healthy individuals, the up-regulated genes were related to metabolic activity, sugar transport, stress tolerance, invasion and pH regulation. *C. albicans* may have a role in root caries progression.
